# The Role of Target and Bystander Cells in Dose-Response Relationship of Radiation-Induced Bystander Effects in Two Cell Lines

**Published:** 2013-02

**Authors:** Shokouhozaman Soleymanifard, Mohammad Taghi Bahreyni Toossi, Ameneh Sazgarnia, Shokoufe Mohebbi

**Affiliations:** 1 Medical Physics Resaerch Center, School of Medicine, Mashhad University of Medical Sciences, Mashhad, Iran; 2 Department of Medical Physics, School of Medicine, Mashhad University of Medical Sciences, Mashhad, Iran

**Keywords:** Dose-response relationship, Micronucleus assay, MRC5, QU-DB, Radiation-induced bystander effect

## Abstract

***Objective(s):*** Radiation effect induced in nonirradiated cells which are adjacent or far from irradiated cells is termed radiation-induced bystander effect (RIBE). Published data on dose-response relationship of RIBE is controversial. In the present study the role of targeted and bystander cells in RIBE dose-response relationship of two cell lines have been investigated.

***Materials and Methods:*** Two cell lines (QU-DB and MRC5) which had previously exhibited different dose-response relationship were selected. In the previous study the two cell lines received medium from autologous irradiated cells and the results showed that the magnitude of damages induced in QU-DB cells was dependent on dose unlike MRC5 cells. In the present study, the same cells irradiated with 0.5, 2 and 4 Gy gamma rays and their conditioned media were transferred to nonautologous bystander cells; such that the bystander effects due to cross-interaction between them were studied. Micronucleus assay was performed to measure the magnitude of damages induced in bystander cells (RIBE level).

***Results:*** QU-DB cells exhibited a dose-dependent response. RIBE level in MRC5 cells which received medium from 0.5 and 2 Gy QU-DB irradiated cells was not statistically different, but surprisingly when they received medium from 4Gy irradiated QU-DB cells, RIBE was abrogated.

***Conclusion:*** Results pertaining to QU-DB and MRC5 cells indicated that both target and bystander cells determined the outcome. Triggering the bystander effect depended on the radiation dose and the target cell-type, but when RIBE was triggered, dose-response relationship was predominantly determined by the bystander cell type.

## Introduction

Signaling network between irradiated cells and their neighbors induces adverse effects in nonirradiated cells. This phenomenon is termed radiation-induced bystander effect (RIBE). Some protective effects have also been observed in bystander cells such as radio or thermoresistance (1), stimulatory growth (2) and differentiation (3). Intercellular communication or transmission of molecular signals via gap junction or by releasing soluble factors into the environment is the main mechanism of RIBE. The nature of factors released by irradiated cells is not well known, but reactive oxygen species (4-8), nitric oxide (4, 9-11), various cytokines and growth factors (9, 12) have been implicated. Since the first observation of RIBE in 1992, numerous studies have been dedicated to this phenomenon, and it has been widely reviewed in the literature (12-19). Although RIBE has been demonstrated in a variety of cell types, tissue models and *in vivo*, Groesser *et al* (20) recently did not succeed to verify a significant bystander effect in six cell lines which received medium from targeted cells irradiated with nitrogen or iron ions. 

In most investigations, bystander effect has been induced by low doses and the results have questioned the validity of simple linear extrapolation of high dose-responses to low dose region, i.e. linear no-threshold model. This model is used to estimate radiation risk at low doses. In fact, at low doses, single cell responses are overcome by responses at tissue levels, such that in some circumstances, RIBE as a result of tissue response predominates the direct effects (19) and consequently enhances or decreases the radiation risk. Therefore, the dose-response relationship is complicated. RIBE has also been demonstrated at high doses, and its contribution to tumor cell killing has been suggested in radiotherapy (21). The role of RIBE in radionuclide therapy is more important, it would compensate the inhomogeneous distribution of radionuclide in tumoral region, and so the nonlabeled cells are also affected. Boyd *et al* (22) tried to find the radionuclides that are more vulnerable to induce RIBE. Despite the benefits of RIBE in tumor cell killing, it increases the adverse effects of normal tissues and secondary cancer probability. Widel *et al* (23) observed a reverse bystander effect which causes nonirradiated bystander cells attenuate damages to irradiated cancerous cells. In another study the same results were observed when authors measured survival fractions of irradiated cells in flasks in which half of the cell populations were shielded (24). If reverse RIBE is demonstrated in more tumors, radiotherapy strategies need to be reevaluated. 

Information relevant to dose-response of RIBE is controversial. Some studies have indicated that the magnitude of damages induced in bystander cells (RIBE level) is independent of dose (4, 8, 11, 25-27). In other studies, RIBE level enhanced as the dose was increased, but it was rapidly saturated at relatively low doses, such that above a certain dose no additional effects would occur (5, 22). On the contrary, in some studies it is evident that RIBE level increases unlimitedly with dose increasing (2, 28-31). It seems that dose-response relationship is governed by the cell type, as in the mentioned studies different cell types have been used. Also previously we observed that when MRC5 and QU-DB cells received medium from autologous irradiated cells, their dose-response relationships were different (32). In the present work RIBEs due to cross-interaction between these two cell lines (QU-DB and MRC5 cells) were studied, and by comparing the results with the previous ones, effort was performed to investigate, whether target or bystander cells determine the dose-response of QU-DB and MRC5 cells. Also in a part of the study, to interpret the main findings, fresh medium was added to the conditioned media extracted from target cells and the effect of medium concentration on QU-DB and MRC5 responses was examined. QU-DB is a human large cell lung carcinoma cell line (33) and MRC5 is a normal lung fibroblast derived from a 14 week old human fetus (34).

## Materials and Methods

*Cell culture and irradiation*

As previously described (32), individual cell lines were cultured in 25cm^2^ flasks (SPL, South Korea) using RPMI-1640 medium (Biosera, England) supplemented with 10% fetal bovine serum (Biosera, England) and antibiotics. Flasks were kept at 37°C in a humidified atmosphere of 95% air and 5% CO_2_. They were divided into two main groups, target and bystander ones. Target groups were irradiated. Irradiation was performed with a ^60^Co teletherapy unit (Theratron, phoenix model, average dose-rate of 70.5cGy/min) at doses of 0.5, 2 and 4 Gy. After irradiation target flasks were returned to the incubator. A group of target cells were sham-irradiated and handled in parallel with irradiated cells. 

*Medium transfer*

Twenty-four hours following irradiation, the culture media of irradiated and sham-irradiated flasks were extracted, filtered through 0.22-µm acetate cellulose filters (Orange Scientific, Belgium), and transferred into nonautologous bystander flasks. As a result, for each cell line there were four bystander subgroups (0 as control, 0.5, 2 and 4Gy bystander subgroups). The first group (control) received medium from sham-irradiated cells. In another experiment the media extracted from 4Gy irradiated cells were diluted with fresh medium and transferred into autologous bystander cells. At the time of medium transfer, cytochalasin B (Sigma Aldrich, USA) was added to the QU-DB and MRC5 bystander flasks to a final concentration which was determined in the previous study (32). 

*Micronucleus assay*

The cytokinesis-block micronucleus assay was used to score micronuclei as an end point of RIBE. For this purpose before performing the main experiments, doubling times of the cell lines were determined as described by Neshasterize *et al* (35). Doubling times of QU-DB and MRC5 cells under the conditions of our laboratory were 16 and 30 hr respectively. Therefore 24 and 45 hr (1.5 doubling time) after addition of cytochalasin B, QU-DB and MRC5 cells were fixed respectively. Based on the concentrations of cytochalasin B which were used, 1.5 doubling time was optimum to allow micronuclei to be expelled into the cytoplasm and consequently appropriate fraction of binucleated cells were prepared. Wang and Coderre also fixed cells after 1.5 doubling time (6). Cell fixation was performed as described previously, briefly QU-DB cells were fixed with methanol; acetic acid taken at a ratio of 3:1 (Merck, Germany) for three times. The protocol used to fix QU-DB cells was not appropriate for MRC5 cells; therefore consistent protocol was used to fix them. They were dried in air and then were fixed only once with absolute methanol which was poured on the bottom of the flasks and let to dry in air. Fixed cells attached to the bottom of the flasks were stained using 10% Geimsa (Merck, Germany) for 5–6 min. The number of cells containing micronuclei (micronucleated cells) per 1000 binucleated cells (MN) was counted at 400× magnification. For accuracy slides were scored twice by one examiner and each time 1000 binucleated cells were scored.

*Statistical analysis*

All data were distributed normally; therefore, parametric tests were used to examine differences between and among the data. One-way analysis of variance and Tukey’s multiple comparison tests were performed to compare bystander subgroups of each cell line. Student’s t-test was used when comparison of two sets of data was considered.

## Results

**Figure 1:**
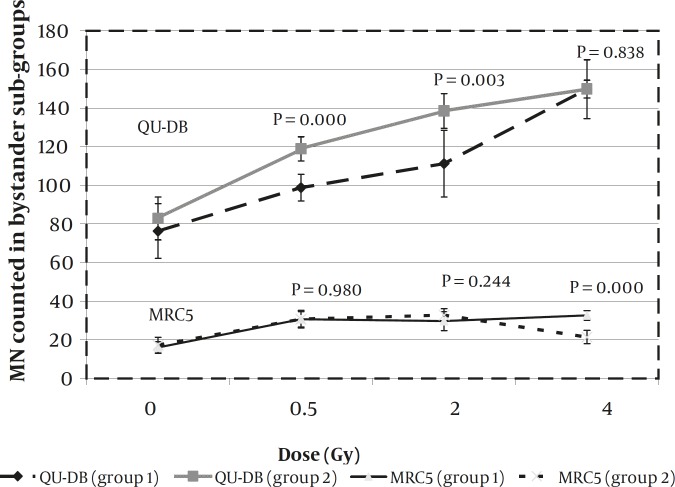
Average number of MN counted in QU-DB and MRC5 bystander subgroups at different doses. Diagrams are plotted to display increasing of MN as a result of increasing radiation dose. P-values at 95% confidence level represent the results of comparing corresponding subgroups of each cell line. Error bars indicate the standard deviation for at least 4 independent experiments. Groups 1 received conditioned medium from autologous irradiated cells, and groups 2 received conditioned medium from nonautologous irradiated cells

Table1 represents the average number of MN counted in QU-DB subgroups that received conditioned medium from autologous (group1) and nonautologous (group 2) irradiated cells. The data of group 1 was obtained in the previous study (32) and are written again to be compared with the new ones. *P*-values represent the significant difference between each subgroup and its control at 95% confidence level. As can be seen, all bystander cells were statistically different than their controls. Also, statistical analysis was performed to compare subgroups with each other. RIBE level in group2 was dose-dependent like in group1; as Tukey’s multiple comparison test indicated that the differences between subgroups were statistically significant (*P < *0.005). Figure 1 shows the data of QU-DB bystander groups. Diagrams are plotted to display variation of RIBE level with dose increasing. 

**Table 1:** Average number of micronucleated cells per 1000 binucleated cells (MN) in groups 1 and 2 of QU-DB bystander cells. Group1 received medium from QU-DB irradiated cells, and group2 received medium from MRC5 irradiated cells

Group	Target cell type	Na	Dose (Gy)	MN (mean) ± SD	Range	*P*-value b
		4	0	76.25 ± 11.06	62-85	-
1	QU-DB	5	0.5	98.80 ± 6.26	95-109	0.005
		5	2	111.20 ± 8.93	102-126	0.000
		5	4	149.75 ± 4.65	145-156	0.000
						
		5	0	82.80 ± 14.13	62-97	-
2	MRC5	7	0.5	118.86± 6.91	109-131	0.001
		8	2	138.50 ± 17.26	124-168	0.000
		6	4	148.33 ± 15.29	126-173	0.000

^a^ N indicates the number of independent experiments performed for specified dose (number of flasks prepared to receive conditioned media from target flasks irradiated with a specified dose)

^b^ *P*-value indicates statistically significant difference at 95% confidence level between every subgroup and control cells

To compare the two bystander groups, corresponding subgroups were compared by student t-test. Corresponding subgroups of the two groups were those that received conditioned medium from target cells (of the two cell lines) irradiated with the same dose. For instance, bystander cells which received medium from 0.5 Gy irradiated QU-DB or MRC5 cells were corresponding. The two groups were statistically different when doses to target cells were 0.5 and 2 Gy, but subgroups which received medium from 4 Gy irradiated cells were not statistically different. Data of this comparison represented by *P*-values is recorded in Figure 1.

Table 2 represents the data obtained from MRC5 bystander cells. Groups are the same as QU-DB cells in Table 1. Cells which received conditioned medium from autologous and nonautologous cells are referred as groups 1 and 2, respectively. Data of group 1 was reported previously (32) and are rewritten to be compared with the new data (group2). *P*-values represent differences between each subgroup and its control. Surprisingly the difference between MRC5 bystander cells which received medium from 4Gy QU-DB irradiated cells and its control was not statistically significant (*P*= 0.179). Statistical analysis was performed to compare subgroups with each other. As previously reported results of the first group indicated that there was no significant difference among the subgroups (*P *> 0.05). In group2, the difference between cells which received conditioned medium from 0.5 and 2Gy irradiated QU-DB cells was not significant (*P =* 0.837). Obviously, differences between the two latter subgroups and cells which received medium from 4 Gy irradiated QU-DB cells were statistically significant (*P < *0.001). Comparison of corresponding subgroups which received medium from autologous and nonautologous target cells, irradiated with the same dose, was performed by Student t-test. Results indicated that there was no significant difference between the two groups at 0.5 and 2Gy. *P-*values are represented in Figure 1.

**Table 2:** Average number of micronucleated cells per 1000 binucleated cells (MN) in groups1 and 2 of MRC5 bystander cells. Group1 received medium from MRC5 irradiated cells, and group2 received medium from QU-DB irradiated cells

Group	Target cell type	Na	Dose (Gy)	MN (mean) ± SD	Range	*P*-value b
		6	0	16.00± 3.03	12-20	-
1	MRC5	5	0.5	30.60 ± 3.91	25-36	0.000
		7	2	29.64 ± 4. 90	21-34	0.000
		5	4	32.60 ± 2.51	29-36	0.000
						
		7	0	17.10±4.08	11-23	-
2	QU-DB	6	0.5	30.67± 4.46	25-38	0.000
		5	2	32.75± 3.30	29-36	0.000
		9	4	21.33 ± 3.46	17-28	0.179

^a^ N indicates the number of independent experiments performed for specified dose (number of flasks prepared to receive conditioned media from target flasks irradiated with a specified dose) ^b^ *P*-value indicates statistically significant difference at 95% confidence level between every subgroup and control cells

**Table 3:** Average number of micronucleated cells per 1000 binucleated cells (MN) in bystander subgroups which received complete or diluted medium from autologous irradiated cells

Cell type	Medium concentration (%)	N a	Dose (Gy)	MN (mean) ± SD	Range
	100	5	4	149.75 ± 4.65	145-156
QU-DB	50	6	4	103.00 ± 4.04	98-109
	100	5	2	111.20 ± 8.93	102-126
					
	100	5	4	32.60 ± 2.51	29-36
MRC5	50	5	4	33.60 ± 5.05	27-41
	25	5	4	32.40 ± 2.90	29-36

a N indicates the number of independent experiments performed for specified dose and specified concentration (number of flasks prepared to receive complete or diluted medium from target flasks irradiated with a specified dose)

Data represented in Table 3 are the results of medium dilution experiments. Conditioned media extracted from 4 Gy irradiated cells were mixed with fresh medium and transferred into only autologous bystander flasks. Medium concentration for QU-DB cells was only 50%, but for MRC5 cells were 50% and 25%. Results indicated that dilution decreased MN frequency of QU-DB cells from 149.75 to 103.00 (*P < *0.0001). Surprisingly, MN frequency of bystander cells which received diluted medium from 4 Gy irradiated cells was not statistically different than MN frequency of cells that received complete medium from 2 Gy irradiated cells (*P =* 0.303). However in MRC5 cells, medium dilution did not affect the number of MN in 50% and 25% diluted groups. Statistical analysis indicated that there was no statistically significant difference between 100% and 50% (*P =* .970), and also between 100% and 25% (*P*= 1.000) concentration groups.

## Discussion

Regarding the pivotal role of cell type in bystander response, the present study was designed to investigate the role of target and bystander cells separately in dose-response relationship of QU-DB and MRC5 cells. In the range of 0.5 to 4 Gy, bystander response of QU-DB was dose-dependent, while MRC5 cells responded to the bystander signals independently of dose (32). To find whether target or bystander cells determine the shape of dose-response curve, both cell lines were irradiated and their conditioned media, unlike the previous study were transferred into nonautologous bystander cells. Then the results were compared with the results of the same bystander cells which received medium from autologous irradiated cells.

As can be seen in Table 1, irrespective of the target cell type, when dose increased, the number of micronucleated cells induced in QU-DB bystander cells increased as well. Also it was observed that the constant response of MRC5 cells in the range of 0.5 to 2 Gy was independent of target cell type (Table 2). Figure 1 shows that MN frequency of QU-DB cells at all conditions is more than MRC5 cells and target cell type cannot change it considerably. The level of bystander response and dose-response relationship of QU-DB and MRC5 cells are predominantly determined by the bystander cell type. However the impact of target cell type cannot be ignored completely. Because RIBE level in QU-DB bystander cells which received medium from 0.5 and 2 Gy irradiated MRC5 cells, though in low scale, were more than those received medium from 0.5 and 2 Gy irradiated QU-DB cells. Also the response of MRC5 bystander cells which received medium from 4Gy irradiated QU-DB cells was lower than those received medium from MRC5 cells irradiated with the same dose. The importance of target cell type in determining the RIBE level has been observed in other studies. Shareef *et al* showed that H460 cells were more capable of inducing RIBE than A549 cells in the same bystander cells (36). Shao *et al observed* that irradiated glioma cells induced a higher level of RIBE than irradiated skin fibroblasts (4). Also, it has been observed that radiation LET influences the target cells ability to induce RIBE (11). Unless the mechanism of RIBE is discovered completely, the role of target cells in RIBE level cannot be explained. Chen *et al* tried to investigate the mechanism of RIBE and the role of mitochondria in the formation and transduction of signals during the early stage of the bystander process (37). They used normal A_L_ cells and mitochondrial DNA-depleted A_L_ cells as target cells. Their results indicated that mitochondrial DNA-depleted cells attenuated RIBE, which indicates that mitochondria play a functional role in bystander effects. Differences between targeted cells mentioned in the above studies are not related to mitochondria; therefore, other molecular pathways as well as mitochondrial downstream pathways may have a functional role in inducing RIBE. 

To interpret dose-dependent response of QU-DB cells (Table 1), it may be suggested that the amount of bystander signals produced by target cells was proportional to dose, such that at a higher dose, more bystander signals were produced by both target cell types, and consequently more bystander responses were exhibited by QU-DB bystander cells. Ryan *et al* (38) observed a linear correlation between signal concentration and bystander response. They used medium transfer technique to induce RIBE in nonirradiated cells. They added fresh culture medium to conditioned media harvested from irradiated cell cultures and designed a series of diluted media. The diluted media were transferred to bystander cells. The results indicated that as the medium concentration was decreased, the RIBE level decreased as well. However, it was not clear whether there is a correlation between dose and signal concentration. To demonstrate a correlation between dose and signal concentration, it is expected that only the cell types that have exhibited dose-dependent RIBE level would be affected by medium dilution. To answer the above question, we designed a similar experiment. Conditioned media extracted from QU-DB and MRC5 target cells were diluted with fresh medium to a concentration of 50% and transferred to autologous bystander cells. The experiment was repeated with 25% medium concentration only for MRC5 cells. The results indicated that as we had predicted QU-DB cells were affected by medium dilution, but the response of MRC5 cells was independent of medium concentration (Table 3). Surprisingly, MN frequency of QU-DB bystander cells which received diluted medium from 4 Gy irradiated cells was not statistically different than MN frequency of the same cells which received complete medium from 2 Gy QU-DB irradiated cells (*P*= 0.303). This observation indicated that the quantity of bystander signals in 2 Gy irradiated cells was equal to the amount of signals exist in the 50% diluted medium extracted from 4 Gy irradiated cells. The impact of reduced dose (2 Gy) was the same as medium dilution. There was a direct correlation between dose and the amount of bystander signals produced by target cells. The constant response of MRC5 cells to different concentrations of medium confirms the above conclusion, because it was expected that the cell types which have not exhibited dose-dependent response would not be affected by medium dilution. However in contrast with our inference, Baskar *et al* revealed while the bystander response of GM637H cells was independent of dose, medium dilution decreased their bystander response (11). To explain dose-dependent response of QU-DB, it is also possible to propose another hypothesis. According to this hypothesis, not only the amount of signals, but also the kinds of signals produced by target cells were changed as the dose increased. So at different doses, different molecular pathways were activated to act as intercellular or intracellular signal carriers, and consequently different levels of RIBE were induced in bystander cells. 

As mentioned above, the number of micronucleated cells counted in MRC5 bystander groups was constant and even decreased when the dose to QU-DB cells rose to 4 Gy. Ryan *et al* have asked whether the saturated response of bystander cells is due to a limited bystander signals induced by target cells or a limited response by bystander cells. (38). The constant response of MRC5 bystander cells was not due to a limited signal production by target cells, since at higher dose, conditioned media harvested from the same target cells were capable of inducing higher RIBE level in QU-DB bystander cells. To explain the constant response of MRC5 bystander cells, it may be hypothesized that the signals received, either low or high, were propagated to a maximum level by bystander cells, and they exhibited the maximum response. The alternative hypothesis is that the bystander signals were not propagated by MRC5 bystander cells, instead MRC5 response was limited to a constant value, and consequently was not dependent on the quantity or type of bystander signals received.

When conditioned medium from 4Gy irradiated QU-DB cells was transferred to MRC5 cells, the bystander effect disappeared and the number of micronucleated cells decreased to control level. It is obvious that bystander factors or signals were present in the conditioned medium from 4Gy irradiated QU-DB cells, as this medium induced RIBE in QU-DB bystander cells. Also, MRC5 cells were affected by the medium from 4Gy irradiated MRC5 cells, and thus both medium and bystander cells exhibited the ability to induce and receive bystander signals, respectively. It seems that the data obtained in the present study is not sufficient to explain this observation, but we can refer to other studies with similar observations. Mackonis *et al* investigated cell survival following spatially modulated beams. They measured cell survival in the shielded and unshielded regions of the modulated fields and compared it with cell survival in uniform control fields. When unshielded regions were irradiated with lethal dose (20 Gy), the survival of shielded regions increased more than uniform control fields. They proposed that the process of death in unshielded regions stimulated a repair mechanism in the viable cells in the shielded regions (39). Gow *et al* used 60Co gamma rays and 20 MeV electrons at doses of 0.5, 5 and 10 Gy with varying dose rates to induce RIBE in HPV-G cells. They observed that survival fraction of the bystander cells decreased when culture media of 0.5 and 5Gy irradiated cells were transferred to bystander cells. When the dose rose to 10 Gy, the RIBE was abolished in either gamma rays or electron particles cases. The authors proposed a negative feedback mechanism as the result of increased signal concentration at 10 Gy (40). The same reasons may be applied to interpret RIBE abrogation in MRC5 cells; however, dose and end-point in this case were different than the corresponding variables in Gow and Mackonis’s investigations. Also, RIBE abrogation in MRC5 cells was target cell type-dependent. 

## Conclusion

In summary, both target and bystander cells determined the final outcome; triggering the RIBE depended on the radiation dose and the target cell-type, but when bystander effect was triggered, RIBE level (damages induced in bystander cells) and dose-response relationship were predominantly determined by the bystander cell type. Data of the dilution medium experiment indicated that there was a direct correlation between dose and bystander signal concentration in QU-DB and MRC5 target cells. These conclusions pertain only to the lines and conditions reported here and to generalize conclusion, the same experiments can be performed with other cell lines which have different dose-response relationships. Abrogation of RIBE in MRC5 cells which received medium from 4 Gy irradiated QU-DB cells may be relevant to dose fractionation in radiation therapy. In radiotherapy, small fractionated doses are designed to irradiate and kill tumor cells and spare normal cells from damages associated with high doses. However, if negative feedback mechanism proposed by Gow *et al* and our observation that intermediate dose (4 Gy) abrogates RIBE are demonstrated as pervasive phenomena, they may be used to explain tissue responses in grid therapy and plan new protocols in radiotherapy. 
